# Body mass index and survival after diagnosis of invasive breast cancer: a study based on the Japanese National Clinical Database—Breast Cancer Registry

**DOI:** 10.1002/cam4.678

**Published:** 2016-02-29

**Authors:** Masaaki Kawai, Ai Tomotaki, Hiroaki Miyata, Takayuki Iwamoto, Naoki Niikura, Keisei Anan, Naoki Hayashi, Kenjiro Aogi, Takanori Ishida, Hideji Masuoka, Kotaro Iijima, Shinobu Masuda, Koichiro Tsugawa, Takayuki Kinoshita, Seigo Nakamura, Yutaka Tokuda

**Affiliations:** ^1^ Department of Breast OncologyMiyagi Cancer CenterNatoriJapan; ^2^ Department of Healthcare Quality AssessmentGraduate School of MedicineThe University of TokyoTokyoJapan; ^3^ Departments of Breast and Endocrine SurgeryOkayama University HospitalOkayamaJapan; ^4^ Departments of Breast and Endocrine SurgeryTokai University School of MedicineIseharaJapan; ^5^ Department of SurgeryKitakyushu Municipal Medical CenterKitakyushuJapan; ^6^ Department of Breast SurgerySt. Luke's International HospitalTokyoJapan; ^7^ Department of Breast SurgeryShikoku Cancer CenterMatsuyamaJapan; ^8^ Department of Surgical OncologyGraduate School of MedicineTohoku UniversitySendaiJapan; ^9^ Sapporo‐kotoni Breast ClinicSapporoJapan; ^10^ Department of Breast OncologyCancer Institute HospitalTokyoJapan; ^11^ Department of PathologyNihon University School of MedicineTokyoJapan; ^12^ Division of Breast and Endocrine SurgeryDepartment of Surgery St. Marianna University School of MedicineKawasakiJapan; ^13^ Department of Breast SurgeryNational Cancer Center HospitalTokyoJapan; ^14^ Division of Breast Surgical OncologyDepartment of SurgeryShowa UniversityTokyoJapan

**Keywords:** Body mass index, breast cancer, menopausal status, subtypes, survival

## Abstract

Few studies have reported the association between body mass index (BMI) and outcome among Asian breast cancer patients. We analyzed data for 20,090 female invasive breast cancer patients who had been followed‐up for a median period of 6.7 years entered in the National Clinical Database–Breast Cancer Registry between 2004 and 2006. We used mainly the WHO criteria for BMI (kg/m^2^) categories; <18.5 (underweight), ≥18.5–<21.8 (reference), ≥21.8–<25, ≥25–<30 (overweight), and ≥30 (obese). We divided normal weight patients into two subgroups because this category includes many patients compared to others. The timing of BMI measurement was not specified. The Cox proportional hazards model and cubic spline regression were used to estimate hazard ratios (HRs) and 95% confidence intervals (CIs). Smoking, alcohol, and physical activity were not controlled. A total of 1418 all‐cause, 937 breast cancer–specific deaths, and 2433 recurrences were observed. Obesity was associated with an increased risk of all‐cause (HR: 1.46; 95% CI: 1.16–1.83) and breast cancer–specific death (HR: 1.47; 95% CI: 1.11–1.93) for all patients, and with all‐cause (HR: 1.47; 95% CI: 1.13–1.92) and breast cancer–specific death (HR: 1.58; 95% CI: 1.13–2.20) for postmenopausal patients. Being underweight was associated with an increased risk of all‐cause death for all (HR: 1.41; 95% CI: 1.16–1.71) and for postmenopausal patients (HR: 1.45; 95% CI: 1.15–1.84). With regard to subtype and menopausal status, obesity was associated with an increased risk of breast cancer–specific death for all cases of luminal B tumor (HR: 2.59; 95% CI: 1.51–4.43; P_heterogeneity_ of Luminal B vs. Triple negative = 0.016) and for postmenopausal patients with luminal B tumor (HR: 3.24; 95% CI: 1.71–6.17). Being obese or underweight is associated with a higher risk of death among female breast cancer patients in Japan.

## Introduction

Obesity defined in terms of body mass index (BMI) is a possible factor affecting the prognosis of patients with breast cancer. A previous meta‐analysis including 43 studies showed that obesity was associated with higher risk of all‐cause or breast cancer–specific death among pre‐ and postmenopausal women 1. A more recent large‐scale meta‐analysis of 82 studies conducted by the World Cancer Research Fund and American Institute for Cancer Research (WCRF/AICR) also showed that obese patients had poorer overall and breast cancer survival, for both pre‐ and postmenopausal patients, and that being underweight was not associated with breast cancer survival, although the latter included only 10 studies 2.

It has been suggested that associations between BMI and outcome in Asians may differ from those in Europe 3. A large‐scale study from Korea including 24,698 breast cancer patients demonstrated significantly lower overall and breast cancer–specific survival and a higher risk of recurrence in patients who were underweight than in those of normal weight, although no conclusion was drawn with regard to any association between overweight/obesity and breast cancer recurrence or death 4. A recent study from Japan suggested that both higher BMI and lower BMI are associated with an increased risk of mortality among breast cancer patients 5. However, the associations between being obese or underweight and survival among breast cancer patients have not been adequately assessed in Asian countries; previous meta‐analyses of Asian patients included only two 1 and seven 2 studies, respectively.

There is biological evidence that breast cancer is a heterogeneous disease 6, 7. There is considerable heterogeneity of breast cancer subtypes, each showing a distinct gene‐expression profile 6, 8. Biological heterogeneity defined by combined estrogen/progesterone receptor (ER/PR) and human epidermal growth factor receptor 2 (HER2) status may imply important differences in tumor etiology and prognosis 9. Thus, assessment of associations between BMI and breast cancer prognosis according to tumor subtypes defined by ER/PR/HER2 may shed further light on this relationship. In fact, several studies have already investigated the effects of tumor subtype defined by ER/PR status 10, 11, 12. A recent meta‐analysis of 21 studies, including the ER/PR status of breast cancer and menopausal status, showed that obesity impacted negatively on both overall and breast cancer survival irrespective of ER/PR and menopausal status 13. However, few studies have addressed the association between obesity and survival of breast cancer patients in terms of ER/PR/HER2 status 14, 15.

In this study, we investigated the relationship between BMI and the risk of all‐cause death and breast cancer–specific death among breast cancer patients in terms of menopausal status and also tumor subtype using a nationwide database in Japan.

## Materials and Methods

### Study subjects, database, and clinical information

The Japanese Breast Cancer Society (JBCS) has maintained the Breast Cancer Registry (BCR) supported by the Public Health Research Foundation (Tokyo). Affiliated institutes have voluntarily provided the BCR with data on newly diagnosed primary breast cancer cases through a web‐based system since 2004 16. The National Clinical Database (NCD) in Japan, which was launched in 2010, is a nationwide prospective web‐based registry linked to the surgical board certification system. Detailed information about the NCD has been published previously 17, 18. In brief, the NCD systematically collects accurate data in order to develop a standardized database for improvement of quality and evaluation of healthcare quality from the standpoint of structure, process, and outcome 17. Detailed information on cancers, such as gastrointestinal, liver, pancreas, thyroid, and breast cancer, is also collected 19. The NCD contains >1.2 million surgical cases collected up to 2011, and approximately 4000 institutions have been participating. The NCD continuously communicates with hospital personnel responsible for data collection through the NCD web‐based data management system, and also consistently performs random site visits to validate the submitted data. Between 2004 and 2011, 238,840 cases were transferred from the JBCS to the NCD for creation of the National Clinical Database—Breast Cancer Registry (NCD‐BCR). For our present study, we used NCD‐BCR data for 53,670 patients who had been newly diagnosed and registered as having breast cancer at 388 institutions between 2004 and 2006 and who were requested to attend for initial follow up at around 8 years after initial diagnosis. An estimate of newly diagnosed female breast cancer cases between 2004 and 2006 is 155,027 20. Newly diagnosed breast cancer cases captured in this registry are 34.6%. Finally, 25,898 patients from 170 institutions were followed up.

Information on patients covering age, sex, height and weight, place of residence, detection method, family history of breast cancer, menopausal status, tumor characteristics, TNM classification, and treatment (chemotherapy, endocrine therapy, radiation therapy) was obtained from the NCD‐BCR. The TNM classification and histological classification were registered according to the UICC staging 21 and WHO classification systems, 22 respectively. Patients who were male (*n* = 231) or of unknown sex (*n* = 1), or who were at stage 0 (*n* = 5546) or IV (*n* = 1355) or unknown stage (*n* = 1349) were excluded, leaving a total of 45,188 patients. Information on ER/PR/HER2 was also obtained from the NCD‐BCR. ER/PR positivity was diagnosed if at least 1% of nuclei in the tumor were immunohistochemically positive for ER or PR. HER2 overexpression was defined as an immunohistochemical score of 3 +  and/or a positive FISH result. Cases were categorized into four subtypes on the basis of their status: luminal A (ER+/PR+/HER2−); luminal B (ER+/PR−/HER2− or ER+/HER2 +); HER2− overexpressing (ER−/PR−/HER2 + ); and triple negative (ER−/PR−/HER2−) 23.

### Ascertainment of exposures and follow up

Body mass index was calculated as weight divided by the square of height (kg/m^2^). Patients whose height or body weight was unknown (*n* = 2582) were excluded, as were those whose age (*n* = 206) and place of residence (*n* = 10) were unknown, leaving a total of 42,390 patients. We categorized BMI into a five‐level variable with reference to the WHO criteria, 24 using a median value of 21.8 between 18.5 and 25.0: <18.5 (underweight), ≥18.5–<21.8 (reference), ≥21.8–<25.0, ≥25.0–<30.0 (overweight), and ≥30 (obese).

**Figure 1:**
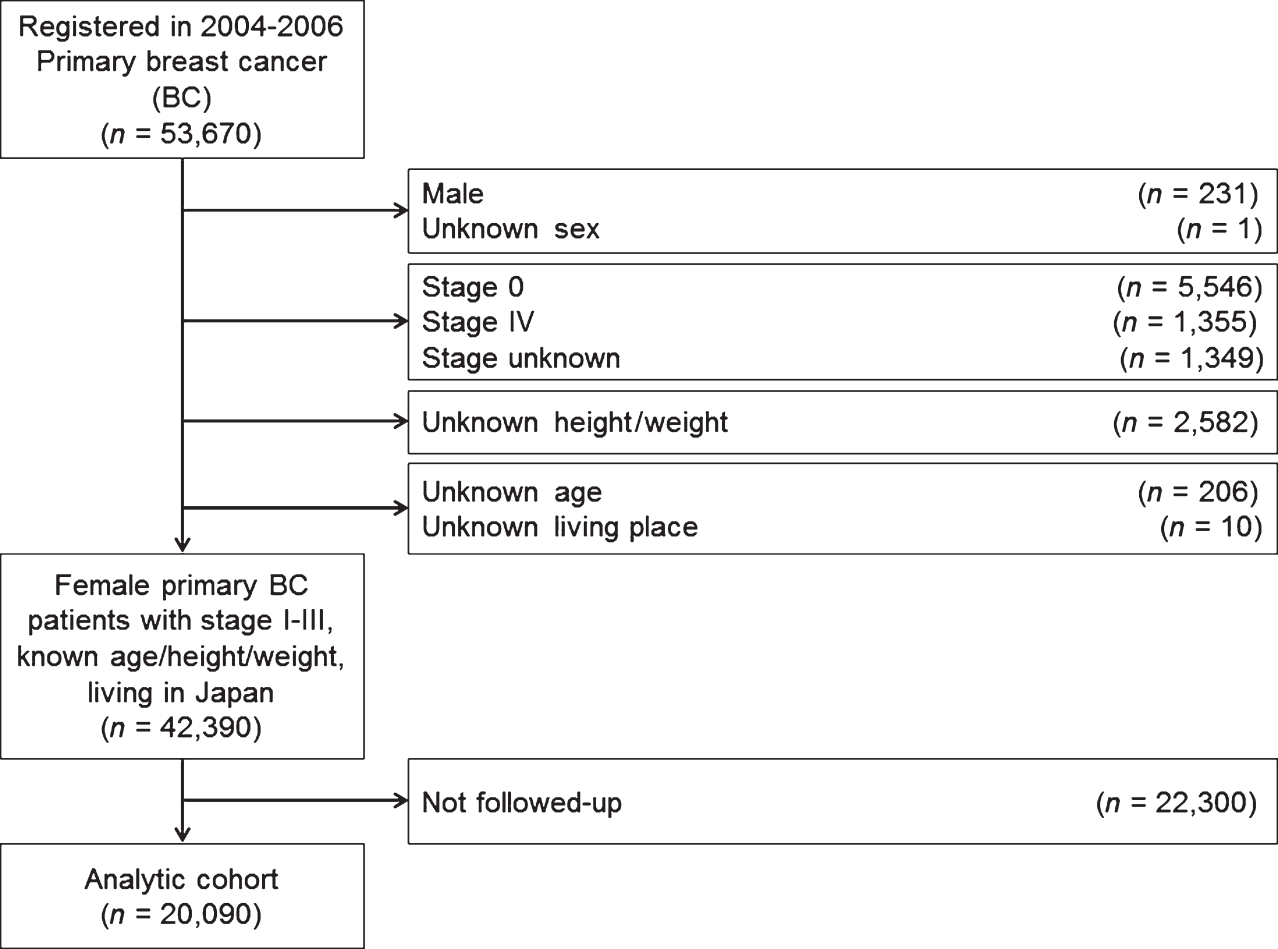
Study flow.

Figure 1 shows a flow diagram of this study. Information on the date of follow up and status (alive, death from breast cancer, death due to causes other than breast cancer, and death due to unknown causes) and the date of recurrence and status (with or without recurrence) were obtained from the NCD‐BCR. During the study period, 20,090 (47.4%) patients were followed up.

### Statistical analysis

The endpoint of our analysis was all‐cause death, breast cancer–specific death, and recurrence. Recurrence included local (conserved breast, chest wall, axillary lymph nodes, and regional lymph nodes) and distant (lung, liver, bone, brain, distant lymph nodes, pleura, and others) metastasis. Survival time was calculated for each patient from the date of first treatment to the date of death, recurrence, or the end of follow up. We used date of first treatment instead of date of diagnosis because the NCD‐BCR does not have date of diagnosis.

The Cox proportional hazards model was used to estimate hazard ratios (HRs) and 95% confidence intervals (CIs) for all‐cause death, breast cancer–specific death, and recurrence in relation to BMI 25. Dose–response relationships were tested by treating each exposure category as a continuous variable and were employed in the Cox model for BMI ≥18.5 because we expected the overall relationship of BMI to each endpoint to be U shaped rather than linear (i.e., we expected patients with BMI <18.5 have higher mortality than the reference category). To evaluate a potential non‐linear relationship between BMI and each endpoint, we applied cubic splines with three knots in settled percentiles (10%, 50%, and 90%) of the distribution to model the possible association 26.

We considered the following variables to be potential confounders: age, place of residence (eastern Japan, western Japan), detection method (self‐detection, screening with symptoms, screening without symptoms, others), family history of breast cancer (no, yes), tumor stage [Stage I, Stage II (IIA/IIB), Stage III (IIIA/IIIB/IIIC)], chemotherapy (no, yes), endocrine therapy (no, yes), radiation therapy (no, yes, unknown), tumor subtype (luminal A, luminal B, HER2, triple negative, others), menopausal status (premenopausal, postmenopausal, unknown), and registration year (2004, 2005, 2006).

Separate analyses were conducted after dividing the patients according to menopausal status and tumor subtype, along with analysis of the patients overall. Menopause was defined as the cessation of menstrual periods for more than 1 year. Menopause resulting from surgery was defined as unknown menopausal status. To evaluate heterogeneity of the associations between BMI and each endpoint across tumor subtypes (Luminal B vs. Luminal A/ HER2− overexpressing/triple negative), interaction terms (BMI * tumor subtypes) were tested.

Results were regarded as significant if the two‐sided *P* values were <0.05. All statistical analyses were performed using the SAS 9.4 (SAS Institute, Cary, NC).

## Results

The patient characteristics are shown in Table 1. During a median follow‐up period of 6.7 years, 1418 all‐cause deaths, 937 breast cancer–specific deaths, and 2433 recurrences were observed. Obese patients were more likely to have an advanced stage of breast cancer, a luminal A tumor, or to have undergone endocrine therapy. Underweight patients were more likely to have self‐detected tumors, and less likely to have undergone chemotherapy.

**Table 1:** Patient characteristics

			BMI									
	Total (*N* = 20,090)		<18.5 (*N* = 1561)		≥18.5–<21.8 (*N* = 6833)		≥21.8–<25 (*N* = 6784)		≥25–<30 (*N* = 4015)		≥30 (*N* = 897)	
	*N*	%	*N*x	%	*N*	%	*N*	%	*N*	%	*N*	%
All‐cause death	1418	7.1	138	8.8	414	6.1	476	7.0	298	7.4	92	10.3
Breast cancer–specific death	937	4.7	73	4.7	287	4.2	323	4.8	191	4.8	63	7.0
Recurrence	2433	12.1	193	12.4	796	11.7	839	11.7	478	11.9	127	14.2
Age (year)												
Mean (SD)	57.3	12.8	54.8	14.7	54.0	12.9	58.6	12.1	60.9	11.9	59.7	11.9
Median	57.0		53.0		53.0		58.0		61.0		60.0	
Follow up												
Median	6.7		6.5		6.7		6.7		6.7		6.6	
Person years	119873.4		8875.8		40967.5		40681.3		24062.4		5286.3	
Living place												
Eastern Japan	9598	47.8	737	47.2	3247	47.5	3260	48.1	1917	47.8	437	48.7
Western Japan	10,492	52.2	824	52.8	3586	52.5	3524	52.0	2098	52.3	460	51.3
Detection method												
Self–detection	14,736	73.4	1219	78.1	4988	73.0	4885	72.0	2948	73.4	696	77.6
Screening with symptom	1203	6.0	81	5.2	441	6.5	402	5.9	235	5.9	44	4.9
Screening without symptom	3131	15.6	175	11.2	1092	16.0	1130	16.7	625	15.6	109	12.2
Others	1020	5.1	86	5.5	312	4.6	367	5.4	207	5.2	48	5.4
Family history of breast cancer												
No	17,078	85.0	1337	85.7	5827	85.3	5762	84.9	3392	84.5	760	84.7
Yes	1761	8.8	132	8.5	589	8.6	604	8.9	364	9.1	72	8.0
Missing	1251	6.2	92	5.9	417	6.1	418	6.2	259	6.5	65	7.3
Tumor stage												
Stage I	8304	41.3	725	46.4	3075	45.0	2765	40.8	1473	36.7	266	29.7
Stage II (IIA/IIB)	9841	49.0	662	42.4	3186	46.6	3376	49.8	2102	52.4	515	57.4
Stage III (IIIA/IIIB/IIIC)	1945	9.7	174	11.2	572	8.4	643	9.5	440	11.0	116	12.9
Treatments												
Chemotherapy												
No	10,638	53.0	889	57.0	3567	52.2	3557	52.4	2154	53.7	471	52.5
Yes	9452	47.1	672	43.1	3266	47.8	3227	47.6	1861	46.4	426	47.5
Endocrine therapy												
No	6524	32.5	539	34.5	2339	34.2	2194	32.3	1220	30.4	232	25.9
Yes	13,566	67.5	1022	65.5	4494	65.8	4590	67.7	2795	69.6	665	74.1
Radiation therapy												
No	10,543	52.5	848	54.3	3408	49.9	3577	52.7	2236	55.7	474	52.8
Yes	9409	46.8	700	44.8	3381	49.5	3161	46.6	1751	43.6	416	46.4
Unknown	138	0.7	13	0.8	44	0.6	46	0.7	28	0.7	7	0.8
Tumor subtypes												
Luminal A	9850	49.0	732	46.9	3252	47.6	3272	48.2	2084	51.9	510	56.9
Luminal B	3988	19.9	327	21.0	1378	20.2	1383	20.4	754	18.8	146	16.3
HER2	1485	7.4	122	7.8	542	7.9	523	7.7	258	6.4	40	4.5
Triple negative	2993	14.9	227	14.5	1064	15.6	1028	15.2	556	13.9	118	13.2
Others	1774	8.8	153	9.8	597	8.7	578	8.5	363	9.0	83	9.3
Menopausal status												
Premenopausal	6785	33.8	696	44.6	3065	44.9	1923	28.4	879	21.9	222	24.8
Postmenopausal	12576	62.6	814	52.2	3524	51.6	4611	68.0	2987	74.4	640	71.4
Unknown (including surgery)	729	3.6	51	3.3	244	3.6	250	3.7	149	3.7	35	3.9
Registered year												
2004	6368	31.7	468	30.0	2157	31.6	2195	32.4	1302	32.4	246	27.4
2005	7199	35.8	561	35.9	2432	35.6	2428	35.8	1434	35.7	344	38.4
2006	6523	32.5	532	34.1	2244	32.8	2161	31.9	1279	31.9	307	34.2

BMI, body mass index.

Table 2 shows the association of BMI with each endpoint. Compared to patients with BMI ≥18.5–<21.8, those with BMI ≥30.0 were shown to have a higher risk of all‐cause death (HR: 1.46; 95% CI: 1.16–1.83; *P* = 0.0012) and breast cancer–specific death (HR: 1.47; 95% CI: 1.11–1.93; *P* = 0.0065). A dose–response relationship was observed between BMI and all‐cause death (P_trend_ = 0.026). Stratification by menopausal status revealed that postmenopausal obese patients had a higher risk of all‐cause death (HR: 1.47; 95% CI: 1.13–1.92; *P* = 0.0045) and breast cancer–specific death (HR: 1.58; 95% CI: 1.13–2.20; *P* = 0.0072). For premenopausal women, our results showed that obesity was associated with non‐significant higher risks of all‐cause death (HR: 1.46; 95% CI: 0.91–2.35) and breast cancer–specific death (HR: 1.34; 95% CI: 0.79–2.27). Underweight patients had a higher risk of all‐cause death among patients as a whole (HR: 1.41; 95% CI: 1.16–1.71; *P* = 0.0005) and among postmenopausal patients (HR: 1.45; 95% CI: 1.15–1.84; *P* = 0.0018).

**Table 2:** HR (95% CI) of each endpoint with BMI overall and by menopausal status

BMI	Cases	Events	All‐cause death			Events	Recurrence			Events	Breast cancer–specific death		
			HR	95% CI	*P*		HR	95% CI	*P*		HR	95% CI	*P*
All													
≥30	897	92	1.46	1.16–1.83	0.0012	127	1.15	0.95–1.39	0.15	63	1.47	1.11–1.93	0.0065
≥25–<30	4015	298	1.04	0.90–1.21	0.58	478	0.97	0.87–1.09	0.61	191	1.03	0.86–1.24	0.75
≥21.8–<25	6784	476	1.02	0.90–1.17	0.74	839	1.02	0.93–1.13	0.68	323	1.03	0.88–1.21	0.72
≥18.5–<21.8	6833	414	Reference^a^			796	Reference^a^			287	Reference^a^		
<18.5	1561	138	1.41	1.16–1.71	0.0005	193	1.09	0.94–1.28	0.26	73	1.16	0.90–1.50	0.27
*P*_*trend*_					0.026				0.6				0.067
Premenopausal													
≥30	222	20	1.46	0.91–2.35	0.12	35	1.21	0.85–1.71	0.29	16	1.34	0.79–2.27	0.28
≥25–<30	879	62	1.10	0.81 –1.49	0.54	121	1.00	0.81 –1.23	0.99	54	1.09	0.78 –1.50	0.63
≥21.8–<25	1923	98	0.90	0.69–1.17	0.44	225	0.91	0.77–1.08	0.26	81	0.86	0.64–1.14	0.29
≥18.5–<21.8	3065	140	Reference^b^		364	Reference^b^			122	Reference^b^		
<18.5	696	32	1.08	0.74–1.59	0.69	72	0.86	0.67–1.11	0.24	23	0.91	0.58–1.43	0.68
*P*_*trend*_					0.21				0.71				0.39
Postmenopausal													
≥30	640	70	1.47	1.13–1.92	0.0045	88	1.15	0.92–1.46	0.23	46	1.58	1.13–2.20	0.0072
≥25–<30	2987	228	1.01	0.84–1.20	0.95	335	0.96	0.83–1.11	0.55	131	1.02	0.80–1.28	0.9
≥21.8–<25	4611	354	1.02	0.87–1.20	0.78	570	1.06	0.93–1.20	0.39	229	1.11	0.91–1.36	0.31
≥18.5–<21.8	3524	264	Reference^b^			414	Reference^b^			156	Reference^b^		
<18.5	814	97	1.45	1.15–1.84	0.0018	113	1.19	0.97–1.47	0.1	45	1.22	0.88–1.71	0.24
*P*_*trend*_					0.11				0.82				0.11

HR, hazard ratio; CI, confidence interval; BMI, body mass index.

a Adjusted by age, living place, detection method, family history of breast cancer, tumor stage, radiation therapy, chemotherapy, endocrine therapy, tumor subtypes, menopausal status, and registered year.

b Adjusted by age, living place, detection method, family history of breast cancer, tumor stage, radiation therapy, chemotherapy, endocrine therapy, tumor subtypes, and registered year.

Table 3 shows the association of BMI with recurrence and breast cancer–specific death according to tumor subtype. Compared to patients with BMI ≥18.5–<21.8, those with BMI ≥30.0 were shown to have a higher risk of breast cancer–specific death (HR: 2.59; 95% CI: 1.51–4.43; *P* = 0.0006; P_heterogeneity_ of Luminal B vs. Triple negative = 0.016) among patients with luminal B tumor. A dose–response relationship was observed between BMI and breast cancer–specific death (P_trend_ = 0.017).

**Table 3:** HR (95% CI) of each endpoint with BMI by tumor subtypes

BMI	Cases	Events	Recurrence			Events	Breast cancer–specific death		
			HR	95% CI	*P*		HR	95% CI	*P*
Luminal A									
≥30	510	50	1.23	0.90–1.68	0.19	17	1.64	0.93–2.90	0.087
≥25–<30	2084	173	1.07	0.87–1.31	0.53	49	1.27	0.84–1.92	0.26
≥21.8–<25	3272	258	1.11	0.92–1.33	0.27	56	1.05	0.71–1.56	0.81
≥18.5–<21.8	3252	221	1.00 (Reference)			46	1.00 (Reference)		
<18.5	732	63	1.24	0.93–1.64	0.14	15	1.39	0.77–2.49	0.27
*P*_*trend*_					0.25				0.075
Luminal B									
≥30	146	26	1.16	0.77–1.75	0.49	18	2.59	1.51–4.43	0.0006
≥25–<30	754	98	0.87	0.68–1.12	0.27	38	1.14	0.75–1.74	0.54
≥21.8–<25	1383	196	1.01	0.83–1.24	0.9	59	1.07	0.73–1.54	0.74
≥18.5–<21.8	1378	194	1.00 (Reference)			56	1.00 (Reference)		
<18.5	327	41	0.97	0.70–1.37	0.88	15	1.32	0.75–2.35	0.34
*P*_*trend*_					0.68				0.017
HER2									
≥30	40	12	1.24	0.68–2.26	0.49	7	1.53	0.68–3.42	0.3
≥25–<30	258	46	0.74	0.52–1.05	0.094	13	0.43	0.23–0.80	0.0077
≥21.8–<25	523	112	0.93	0.72–1.22	0.61	43	0.73	0.48–1.10	0.13
≥18.5–<21.8	542	114	1.00 (Reference)			50	1.00 (Reference)		
<18.5	122	25	0.95	0.61–1.47	0.8	12	0.99	0.52–1.89	0.98
*P*_*trend*_					0.32				0.097
Triple negative									
≥30	118	29	1.09	0.74–1.62	0.67	18	1.11	0.67–1.84	0.68
≥25–<30	556	114	0.95	0.75–1.20	0.66	72	1.03	0.77–1.39	0.84
≥21.8–<25	1028	230	1.08	0.89–1.31	0.44	145	1.15	0.90–1.48	0.26
≥18.5–<21.8	1064	200	1.00 (Reference)			112	1.00 (Reference)		
<18.5	227	50	1.15	0.84–1.57	0.39	24	0.97	0.62–1.51	0.89
*P*_*trend*_					0.97				0.65
Luminal B versus Luminal A – P_heterogeneity_ of trends					0.25				0.7
Luminal B versus HER2 – P_heterogeneity_ of trends					0.56				0.0062
Luminal B versus Triple negative – P_heterogeneity_ of trends					0.79				0.059
Luminal B versus Luminal A – P_heterogeneity_ of BMI ≥30					0.77				0.25
Luminal B versus HER2 – P_heterogeneity_ of BMI ≥30					0.86				0.29
Luminal B versus Triple negative – P_heterogeneity_ of BMI ≥30					0.82				0.016

Adjusted by age, living place, detection method, family history of breast cancer, tumor stage, radiation therapy, chemotherapy, endocrine therapy, menopausal status, and registered year.

HR, hazard ratio; CI, confidence interval; BMI, body mass index.

Stratification by menopausal status among patients with luminal B tumor (Table 4) revealed that postmenopausal obese patients had a higher risk of breast cancer–specific death (HR: 3.24; 95% CI: 1.71–6.17; *P* = 0.0003). A dose–response relationship was observed between BMI and breast cancer–specific death (P_trend_ = 0.022).

**Table 4:** HR (95% CI) of each endpoint with BMI by menopausal status among luminal B tumor

BMI	Cases	Events	Recurrence			Events	Breast cancer–specific death		
			HR	95% CI	*P*		HR	95% CI	*P*
Premenopausal									
≥30	30	8	1.41	0.67–3.01	0.37	4	1.95	0.63–6.10	0.25
≥25–<30	114	17	0.97	0.57–1.66	0.91	7	1.07	0.45–2.55	0.87
≥21.8–<25	283	42	0.93	0.63–1.37	0.73	12	0.90	0.44–1.84	0.77
≥18.5–<21.8	481	73	Reference			23	Reference		
<18.5	100	12	0.83	0.45–1.54	0.56	3	0.74	0.22–2.49	0.62
*P*_*trend*_					0.67				0.47
Postmenopausal									
≥30	109	18	1.09	0.66–1.80	0.72	14	3.24	1.71–6.17	0.0003
≥25–<30	613	76	0.81	0.61–1.09	0.16	29	1.19	0.71–1.99	0.5
≥21.8–<25	1054	147	1.02	0.80–1.30	0.89	46	1.27	0.80–2.02	0.31
≥18.5–<21.8	847	118	Reference			31	Reference		
<18.5	215	27	0.95	0.63–1.45	0.81	10	1.39	0.68–2.86	0.37
*Ptrend*					0.35				0.022

HR, hazard ratio; CI, confidence interval; BMI, body mass index.

Adjusted by age, living place, detection method, family history of breast cancer, tumor stage, radiation therapy, chemotherapy, endocrine therapy, and registered year.

**Figure 2:**
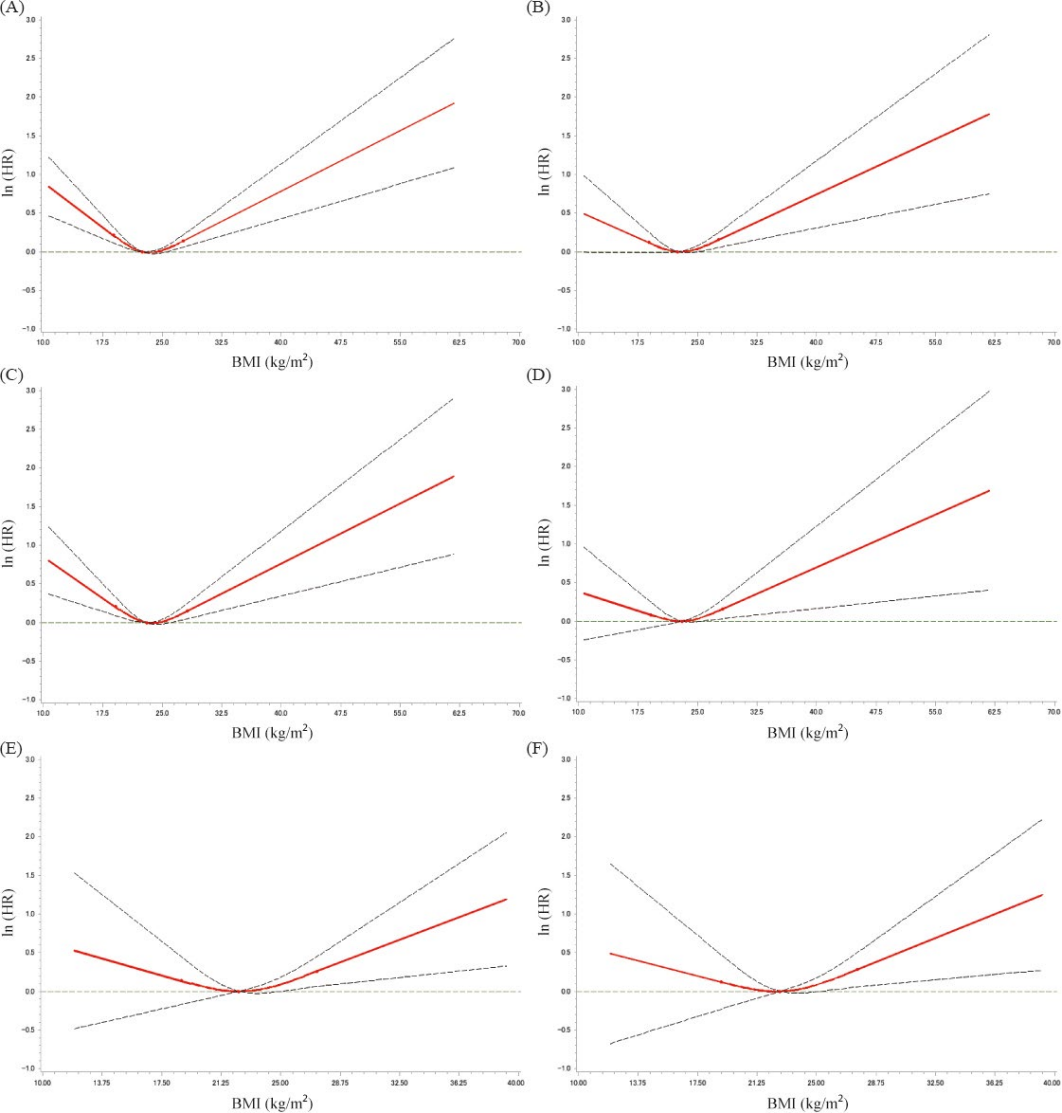
HR (ln of HR) and the corresponding 95% confidence intervals (CIs), using multivariate restricted cubic splines between body mass index (BMI) and each endpoint: (A) all‐cause death for all, (B) breast cancer–specific death for all, (C) all‐cause death for postmenopausal, (D) breast cancer–specific death for postmenopausal, (E) breast cancer–specific death for all with luminal B tumor, and (F) breast cancer–specific death for postmenopausal with luminal B tumor. The solid line and dash lines indicate HR and 95% CI.

Figure 2 shows HR and the corresponding 95% CI of multivariate‐restricted cubic splines between BMI and each endpoint. A dose–response relationship was observed between BMI and all‐cause death, higher BMI and breast cancer–specific death (Fig. 2A and B) overall. Among postmenopausal patients a dose–response relationship was observed between BMI and all‐cause death, higher BMI and breast cancer–specific death (Fig. 2C and D). Among patients with luminal B tumor, a dose–response relationship was observed between higher BMI and breast cancer–specific death overall (Fig. 2E) and postmenopausal patients (Fig. 1F).

## Discussion

Our present study demonstrated that being obese or underweight was associated with an increased risk of death overall, especially for postmenopausal patients. In terms of tumor subtype and menopausal status, obesity was associated with an increased risk of death in patients with luminal B tumor and in patients who were postmenopausal. The association between BMI and survival among breast cancer patients has not been adequately addressed in Asian countries 1, 2. Our study is therefore of importance in that a nationwide database in Japan has been analyzed for the first time in a prospective setting, involving a large number of breast cancer patients stratified according to tumor subtype and menopausal status.

A meta‐analysis including 213,075 breast cancer patients with 41,477 deaths (23,182 from breast cancer) reported that the relative risk (RR) of total mortality for obese patients was 1.41 (95% CI: 1.29–1.53) and that of breast cancer mortality was 1.35 (95% CI: 1.24–1.47) in comparison with patients of normal weight 2. That study also revealed that the RR of total mortality for obese patients was 1.75 (95% CI: 1.26–2.41) among those who were premenopausal and 1.34 (95% CI: 1.18–1.53) for those who were postmenopausal, whereas the RR of breast cancer mortality was 1.50 (95% CI: 1.13–2.00) for premenopausal women and 1.34 (95% CI: 1.21–1.48) for postmenopausal women in comparison with women of normal weight 2. Our present results are consistent with these, showing that obesity was associated with a higher risk of all‐cause death and breast cancer–specific death for the patients overall and for postmenopausal patients. For premenopausal women, our present results demonstrated that obesity was associated with a non‐significant higher risk of all‐cause death and breast cancer–specific death. One possible reason for this relationship may have been the slightly higher proportion of obese patients with advanced‐stage breast cancer. Therefore, we attempted to analyze the data for Stage I breast cancer alone. However, this yielded almost the same results (Table 5). We hypothesized a reason for a slightly higher proportion of obese patients with advanced‐stage cancer. This might be due to the development of more aggressive tumors rather than screening behavior. The proportion of TNBC, an aggressive type of tumor, in overweight or obese women was lower than others.

**Table 5:** HR (95% CI) of each endpoint with BMI by Stage I or I + II overall and by menopausal status

	Cases	Events	All‐cause death			Events	Recurrence			Events	Breast cancer–specific death		
			HR	95% CI	*P*		HR	95% CI	*P*		HR	95% CI	*P*
Stage I													
All													
≥30	266	17	2.85	1.66–4.92	0.0002	17	1.69	1.02–2.81	0.041	7	3.38	1.46–7.83	0.0045
≥25–<30	1473	52	1.30	0.89–1.89	0.18	76	1.14	0.86–1.51	0.38	20	1.42	0.79–2.55	0.24
≥21.8–<25	2765	79	1.17	0.83–1.64	0.37	140	1.10	0.87–1.39	0.43	33	1.32	0.79–2.20	0.29
≥18.5–<21.8	3075	62	1.00 (Reference)^a^			155	1.00 (Reference)^a^			28	1.00 (Reference)^a^		
<18.5	725	27	1.90	1.21–2.99	0.0056	46	1.31	0.94–1.82	0.11	8	1.36	0.62–3.00	0.44
*P*_*trend*_					0.0026				0.081				0.017
Premenopausal													
≥30	59	1	3.69	0.46–29.63	0.22	0	–	–	–	0	–	–	–
≥25–<30	276	6	2.46	0.89–6.76	0.082	16	1.54	0.89–2.68	0.13	4	2.60	0.75–9.04	0.13
≥21.8–<25	765	13	2.25	0.99–5.07	0.052	38	1.25	0.83 –1.86	0.29	10	2.85	1.07–7.60	0.037
≥18.5–<21.8	1407	11	1.00 (Reference)^b^			67	1.00 (Reference)^b^			7	1.00 (Reference)^b^		
<18.5	344	2	0.75	0.17–3.43	0.71	19	1.10	0.66–1.83	0.73	1	0.56	0.07–4.60	0.59
*P*_*trend*_					0.034				0.51				0.12
Postmenopausal													
≥30	198	14	2.43	1.33–4.44	0.0038	16	2.16	1.26–3.72	0.0052	6	3.12	1.23–7.91	0.017
≥25–<30	1145	45	1.15	0.77–1.74	0.5	57	1.04	0.74 –1.46	0.82	15	1.15	0.58–2.27	0.7
≥21.8–<25	1906	64	1.03	0.70–1.50	0.89	96	1.05	0.78 –1.42	0.74	22	1.03	0.55–1.93	0.92
≥18.5–<21.8	1571	49	1.00 (Reference)^b^			84	1.00 (Reference)^b^			19	1.00 (Reference)^b^		
<18.5	354	22	1.85	1.11–3.07	0.018	23	1.29	0.81 –2.04	0.29	5	1.29	0.48–3.48	0.61
*P*_*trend*_					0.041				0.11				0.14
Stage I + II													
All													
≥30	781	63	1.69	1.29–2.23	0.0002	85	1.27	1.01–1.60	0.039	39	1.83	1.29–2.60	0.0007
≥25–<30	3575	194	1.04	0.87–1.25	0.67	332	1.03	0.90–1.18	0.69	110	1.07	0.84–1.37	0.56
≥21.8–<25	6141	340	1.10	0.94–1.29	0.23	622	1.10	0.98–1.24	0.092	213	1.20	0.98–1.46	0.079
≥18.5–<21.8	6261	283	1.00 (Reference)^a^			596	1.00 (Reference)^a^			181	1.00 (Reference)^a^		
<18.5	1387	87	1.41	1.11–1.80	0.0049	138	1.10	0.91–1.32	0.34	39	1.08	0.76–1.52	0.68
*P*_*trend*_					0.014				0.11				0.014
Premenopausal													
≥30	190	10	1.71	0.88–3.30	0.11	20	1.17	0.74–1.84	0.51	9	1.79	0.89–3.60	0.1
≥25– <30	766	37	1.47	1.00–2.17	0.05	81	1.15	0.89–1.47	0.28	32	1.51	1.00–2.30	0.052
≥21.8–<25	1735	61	1.11	0.80–1.54	0.53	166	1.04	0.86–1.26	0.7	50	1.09	0.76–1.56	0.65
≥18.5–<21.8	2844	89	1.00 (Reference)^b^			276	1.00 (Reference)^b^			76	1.00 (Reference)^b^		
<18.5	643	19	0.97	0.59–1.59	0.9	54	0.83	0.62–1.11	0.2	11	0.65	0.35–1.23	0.19
*P*_*trend*_					0.025				0.23				0.025
Postmenopausal													
≥30	558	51	1.66	1.22–2.27	0.0014	63	1.37	1.04–1.80	0.024	29	1.90	1.25–2.88	0.0026
≥25–<30	2677	153	0.96	0.77–1.19	0.7	238	1.00	0.84–1.19	0.99	75	0.98	0.72–1.32	0.88
≥21.8–<25	4172	260	1.07	0.89–1.29	0.48	421	1.12	0.97–1.30	0.13	153	1.26	0.98–1.63	0.073
≥18.5–<21.8	3191	186	1.00 (Reference)^b^			306	1.00 (Reference)^b^			98	1.00 (Reference)^b^		
<18.5	697	62	1.44	1.08–1.93	0.013	79	1.26	0.98–1.62	0.067	26	1.33	0.86–2.05	0.2
*P*_*trend*_					0.14				0.23				0.13

HR, hazard ratio; CI, confidence interval; BMI, body mass index.

a Adjusted by age, living place, detection method, family history of breast cancer, radiation therapy, chemotherapy, endocrine therapy, tumor subtypes, menopausal status, and registered year.

b Adjusted by age, living place, detection method, family history of breast cancer, radiation therapy, chemotherapy, endocrine therapy, tumor subtypes, and registered year.

Our present study demonstrated that underweight patients had an increased risk of all‐cause death, among both the patients overall and those who were postmenopausal. A previous meta‐analysis of 10 studies had shown that being underweight had no association with breast cancer survival 2. Also a large study of Korean breast cancer patients had shown that underweight patients were at a significantly higher risk of all‐cause death (HR: 1.48; 95% CI: 1.15–1.90) 4. Underweight patients might have included undernourished patients, especially among postmenopausal women, as well as properly nourished, naturally lean patients. In patients showing chronic undernutrition, cytokine reactions and subsequent activation of the immune system are compromised 27. This may have partly contributed to the increased risk of all‐cause death among underweight, postmenopausal women. Another reason for the association between being underweight and the higher risk of all‐cause death might have been the slightly higher proportion of patients with advanced‐stage breast cancer. Therefore, we attempted to analyze the data by omitting cases of advanced breast cancer. However, this yielded almost the same results (Table 5).

A few studies have reported the association between BMI and survival of breast cancer patients with combined ER/PR/HER2 status 14, 15. One study found that a higher BMI was associated with shorter disease‐free survival in postmenopausal patients, but no independent effect of any specific subtype was observed 14. The other study showed that patients with ER–/HER2 + tumors showed significantly worse overall survival and that a higher proportion of obese patients had distant metastases 15. In our present study, an association of obesity with poorer outcome was seen in patients with luminal B tumors overall and among postmenopausal patients. Patients with luminal B tumors had a poorer prognosis than those with luminal A tumors 6 and were usually recommended to undergo endocrine therapy and chemotherapy 23. Obese and older breast cancer patients tend to show poorer survival because of suboptimal chemotherapy resulting from comorbidities and chemotherapy dose reduction due to concerns about toxicity 28. Differences in trastuzumab treatment might modify the association of obesity with breast cancer survival in patients with HER2‐positive breast cancer. Among the patients receiving trastuzumab and chemotherapy, 29 those who are obese might show poorer survival than normal weight patients because of more severe trastuzumab‐induced cardiotoxicity 30. Obesity is also associated with poorer survival after endocrine therapy in breast cancer patients 31. In postmenopausal obese patients, higher synthesis of peripheral estrogen in adipose tissue 32, 33, 34 is the most likely mechanism responsible for the higher risk of breast cancer–specific death 35. A recent systematic review reported that obesity was associated with decreased efficacy of endocrine therapy in postmenopausal patients with hormone receptor‐positive tumors 36. Suboptimal endocrine therapy and chemotherapy might explain the poorer outcome of postmenopausal obese patients with luminal B tumors. Further studies will be needed to clarify these associations.

There was increasing evidence that a specific BMI reflects a higher percentage of body fat among Asian populations at a given BMI than do white or European 37. A previous study in Japan suggested that BMI ≥25 adequately specifies complication, 38 where the prevalence and degree of obesity remain mild 39. A WHO Expert Consultation panel in 2002 proposed BMI cut‐off points for Asians for policy and intervention strategies; <18.5 (underweight), ≥18.5–<23 (reference), ≥23–<27.5 (increased risk), and ≥27.5 (high risk). Table 6 shows the association of BMI with each endpoint by this cut offs overall and by menopausal status. Those with BMI ≥27.5 were not shown to have a higher risk of all‐cause death, but shown to have a higher risk of breast cancer–specific death (HR: 1.24; 95% CI: 1.01–1.52; *P* = 0.0038). A dose–response relationship was observed between BMI and breast cancer–specific death (P_trend_ = 0.048). Postmenopausal patients with BMI ≥27.5 did not have a higher risk of all‐cause death, but have a higher risk of breast cancer–specific death (HR: 1.30; 95% CI: 1.02–1.65; *P* = 0.035). A dose–response relationship was observed between BMI and breast cancer–specific death (P_trend_ = 0.04). There might be some kind of metabolic reserve which is not protective at high levels of obesity for all‐cause death. BMI cut‐off points for Asians might not be useful for policy, and intervention strategies in this cohort as BMI of ≥18.5–<25 were already recommended to maintain a healthy condition 40. The WHO Expert Consultation also recommended to use all categories for reporting purposes with a view to facilitating international comparisons whenever possible 37.

**Table 6:** HR (95% CI) of each endpoint with BMI cut offs for Asian populations overall and by menopausal status

BMI	Cases	Events	All‐cause death			Events	Recurrence			Events	Breast cancer–specific death		
			HR	95% CI	*P*		HR	95% CI	*P*		HR	95% CI	*P*
All													
≥27.5	2130	179	1.16	0.98–1.37	0.09	282	1.05	0.92–1.20	0.46	125	1.24	1.01–1.52	0.038
≥23–<27.5	6685	485	1.04	0.92–1.17	0.55	797	0.97	0.88–1.06	0.46	320	1.05	0.91–1.22	0.49
≥18.5–<23	9714	616	1.00 (Reference)^a^			1161	1.00 (Reference)^a^			419	1.00 (Reference)^a^		
<18.5	1561	138	1.40	1.16–1.69	0.0004	193	1.08	0.93–1.26	0.33	73	1.17	0.91–1.50	0.23
*P*_*trend*_					0.11				0.77				0.048
Premenopausal													
≥27.5	474	36	1.20	0.84–1.73	0.32	68	1.06	0.82–1.37	0.67	31	1.19	0.80–1.76	0.39
≥23–<27.5	1669	101	1.03	0.81–1.32	0.81	209	0.94	0.80–1.11	0.48	84	0.99	0.76–1.29	0.93
≥18.5–<23	3946	183	1.00 (Reference)^b^			468	1.00 (Reference)^b^			158	1.00 (Reference)^b^		
<18.5	696	32	1.11	0.76–1.62	0.6	72	0.87	0.68–1.11	0.26	23	0.94	0.60–1.46	0.78
*P*_*trend*_					0.35				0.94				0.5
Postmenopausal													
≥27.5	1575	140	1.16	0.96–1.41	0.13	204	1.06	0.91 –1.25	0.45	92	1.30	1.02–1.65	0.035
≥23–<27.5	4753	364	1.03	0.89–1.18	0.74	545	0.97	0.86 –1.08	0.54	224	1.09	0.91–1.31	0.36
≥18.5–<23	5434	412	1.00 (Reference)^b^			658	1.00 (Reference)^b^			246	1.00 (Reference)^b^		
<18.5	814	97	1.45	1.16–1.81	0.0011	113	1.16	0.95 –1.42	0.14	45	1.22	0.89–1.67	0.23
*P*_*trend*_					0.19				0.71				0.04

HR, hazard ratio; CI, confidence interval; BMI, body mass index.

a Adjusted by age, living place, detection method, family history of breast cancer, tumor stage, radiation therapy, chemotherapy, endocrine therapy, tumor subtypes, menopausal status, and registered year.

b Adjusted by age, living place, detection method, family history of breast cancer, tumor stage, radiation therapy, chemotherapy, endocrine therapy, tumor subtypes, and registered year.

Several limitations of our study should be considered. First, due to the timing of weight measurement, exposure might have been misclassified to some degree. This might be important, as there are some concerns regarding the adverse prognostic implications of a change in BMI after diagnosis of breast cancer 41. A recent review describing the association between weight change and breast cancer prognosis concluded that the existing data are conflicting 42. A meta‐analysis found no significant difference between the timing of BMI measurement and breast cancer outcome 2. Second, there was a relatively low rate of follow up during the study period. Follow up was requested from the NCD and performed by each of the institutions participating in the NCD‐BCR, but was not mandatory. We compared the background of patients who were followed up and those who were not, but found no apparent difference between the two groups (Table S1). The results of our study were almost consistent with those of the previous large‐scale meta‐analysis of qualified studies conducted by the WCRF/AICR 2. Third, our study included no information on comorbidity. Both obese and underweight patients are thought to have a higher risk of comorbid conditions. Fourth, Japan is ethnically homogeneous, and the patients included in our study were all female patients living in Japan. Therefore, the external validity of our results will need to be considered cautiously. Fifth, our study included no information on body composition. The relation between BMI and lean or fat mass may differ between people, but BMI cannot distinguish lean mass from body fat distribution. There have been several studies investigating between body fatness and survival in breast cancer patients. Two studies found negative association between body fat and survival, 43, 44 but others did not 45, 46. Sixth, we do not have key confounders: smoking, alcohol, and physical activity. This might cause bias. Smoking rate in 2015 was 10.6% and alcohol consumption rate in 2005 was 8.0% 47 among Japanese women, which were lower than other countries. Recent large studies from Korea also do not have items of smoking, alcohol, and physical activity 4, 48.

Among breast cancer patients living in Japan, being obese or underweight is associated with a higher risk of all‐cause death, especially in postmenopausal patients. There is some suggestion that postmenopausal obese patients with luminal B tumors have a poorer prognosis. A few studies have addressed the association between underweight and outcome of breast cancer patients in terms of ER/PR/HER2 status 1, 2, 14, 15. As higher and lower BMI are directly related to mortality, 49 it is important for breast cancer patients to maintain an appropriate body weight for height.

## Conflict of Interest

The authors have no conflict of interest.

## Supporting information


**Table S1.** Patient characteristics.
