# Supplementary material for: Body mass index and survival after diagnosis of invasive breast cancer: a study based on the Japanese National Clinical Database—Breast Cancer Registry
Source: Cancer Med. 2016 Feb 29;5(6):1328–40. doi: 10.1002/cam4.678 (PMC4924391; doi:10.1002/cam4.678)
Supplement: Supplementary file 1 — Table S1. Patient characteristics. [file CAM4-5-1328-s001.docx]

**Supplementary table.** Patient characteristics.

|  |  |  |  | Followed-up | |  | Not followed-up | |
| --- | --- | --- | --- | --- | --- | --- | --- | --- |
|  |  |  |  | (N = 20,090) | |  | (N = 22,300) | |
|  |  |  |  | N | % |  | N | % |
| Age (year) | | Mean (SD) |  | 57.3 | 12.8 |  | 57.2 | 12.9 |
|  |  | Median |  | 57.0 |  |  | 57.0 |  |
| Detection method | | Self-detection |  | 14,736 | 73.4 |  | 16,614 | 74.5 |
|  |  | Screening with symptom |  | 1,203 | 6.0 |  | 918 | 4.1 |
|  |  | Screening without symptom |  | 3,131 | 15.6 |  | 3,674 | 16.5 |
|  |  | Others |  | 807 | 4.0 |  | 997 | 4.5 |
|  |  | Missing |  | 213 | 1.1 |  | 97 | 0.4 |
| Family history of breast cancer | | No |  | 17,078 | 85.0 |  | 19,421 | 87.1 |
|  |  | Yes |  | 1,761 | 8.8 |  | 1,779 | 8.0 |
|  |  | Missing |  | 1,251 | 6.2 |  | 1,100 | 4.9 |
| Tumor stage |  | Stage I |  | 8,304 | 41.3 |  | 9,518 | 42.7 |
|  |  | Stage II (IIA/IIB) |  | 9,841 | 49.0 |  | 10,524 | 47.2 |
|  |  | Stage III (IIIA/IIIB/IIIC) |  | 1,945 | 9.7 |  | 2,258 | 10.1 |
| Treatments | |  |  |  |  |  |  |  |
|  | Chemotherapy | No |  | 10,638 | 53.0 |  | 11,563 | 51.9 |
|  |  | Yes |  | 9,452 | 47.1 |  | 10,737 | 48.2 |
|  | Endocrine therapy | No |  | 6,524 | 32.5 |  | 6,665 | 29.9 |
|  |  | Yes |  | 13,566 | 67.5 |  | 15,635 | 70.1 |
|  | Radiation therapy | No |  | 10,543 | 52.5 |  | 11,532 | 51.7 |
|  |  | Yes |  | 9,409 | 46.8 |  | 10,594 | 47.5 |
|  |  | Missing |  | 138 | 0.7 |  | 174 | 0.8 |
| Tumor subtypes | | Luminal A |  | 9,850 | 49.0 |  | 11,026 | 49.4 |
|  |  | Luminal B |  | 3,988 | 19.9 |  | 4,035 | 18.1 |
|  |  | HER2 |  | 1,485 | 7.4 |  | 1,591 | 7.1 |
|  |  | Triple negative |  | 2,993 | 14.9 |  | 2,933 | 13.2 |
|  |  | Others |  | 1,774 | 8.8 |  | 2,715 | 12.2 |
| Menopausal status | | Premenopausal |  | 6,785 | 33.8 |  | 7,746 | 34.7 |
|  |  | Postmenopausal |  | 12,576 | 62.6 |  | 14,064 | 63.1 |
|  |  | Missing (including surgery) |  | 729 | 3.6 |  | 490 | 2.2 |
| Registered year | | 2004 |  | 6,368 | 31.7 |  | 5,193 | 23.3 |
|  |  | 2005 |  | 7,199 | 35.8 |  | 7,738 | 34.7 |
|  |  | 2006 |  | 6,523 | 32.5 |  | 9,369 | 42.0 |
